# Ulinastatin alleviates early brain injury after traumatic brain injury by inhibiting oxidative stress and apoptosis

**DOI:** 10.1590/acb370108

**Published:** 2022-04-20

**Authors:** Xiaoyan Feng, Weiwei Ma, Junhui Chen, Wei Jiao, Yuhai Wang

**Affiliations:** 1 MM. Department of Neurosurgery – 904th Hospital of Joint Logistic Support Force of PLA – Wuxi Clinical College of Anhui Medical University – Wuxi, China.; 2 BS. Department of Neurosurgery – 904th Hospital of Joint Logistic Support Force of PLA – Wuxi Clinical College of Anhui Medical University – Wuxi, China.; 3 MD. Department of Neurosurgery – 904th Hospital of Joint Logistic Support Force of PLA – Wuxi Clinical College of Anhui Medical University – Wuxi, China.; 4 PhD. Department of Neurosurgery – 904th Hospital of Joint Logistic Support Force of PLA – Wuxi Clinical College of Anhui Medical University – Wuxi, China.

**Keywords:** Brain Injuries, Traumatic, Oxidative Stress, Apoptosis, Mice

## Abstract

**Purpose:**

Traumatic brain injury (TBI) remains a major public health problem and cause of death. Ulinastatin (UTI), a serine protease inhibitor, has been reported to have an anti-inflammatory effect and play a role in immunoregulation and organ protection by reducing reactive oxygen species (ROS) production, oxidative stress and inflammation. However, the neuroprotective of UTI in TBI has not been confirmed. Therefore, this study aimed to investigate the neuroprotection and potential molecular mechanisms of UTI in TBI-induced EBI in a C57BL/6 mouse model.

**Methods:**

The neurological score and brain water content were evaluated. Enzyme-linked immunosorbent assay was used to detect neuroinflammatory cytokine levels, ROS and malondialdehyde detection to evaluate oxidative stress levels, and TUNEL staining and western blotting to examine neuronal damages and their related mechanisms.

**Results:**

Treatment with UTI markedly increased the neurological score; alleviated brain oedema; decreased the inflammatory cytokine tumour necrosis factor a, interleukin-1β (IL-1β), IL-6 and nuclear factor kappa B (NF-kB) levels; inhibited oxidative stress; decreased caspase-3 and Bax protein expressions; and increased the Bcl-2 levels, indicating that UTI-mediated inhibition of neuroinflammation, oxidative stress and apoptosis ameliorated neuronal death after TBI. The neuroprotective capacity of UTI is partly dependent on the TLR4/NF-kB/p65 signalling pathway.

**Conclusions:**

Therefore, this study reveals that UTI improves neurological outcomes in mice and reduces neuronal death by protecting against neural neuroinflammation, oxidative stress and apoptosis.

## Introduction

Traumatic brain injury (TBI) remains a major public health problem and cause of death and disability that imposes a substantial economic burden worldwide. Its incidence is high in low- and middle-income countries^1^^-^ 3 and is rapidly increasing due to the significant increase in road traffic collisions, including motor vehicle accidents and the elderly population’s fall injury^3^. An increasing number of randomised controlled trials have been conducted in recent years, which thereby substantially improved the long-term outcomes; however, no significant benefits were observed after drug interventions^2^^-^ 8. Therefore, studies aimed to further clarify the pathophysiological mechanisms of TBI and identify new and effective pharmacological intervention targets are very important and necessary.

The pathophysiology of TBI includes various physiological changes and mainly involves primary and secondary brain injuries, which result in neuronal death, neurological deficits and mortality after TBI^9^. Primary brain injuries frequently result in brain tissue disorganisation, intracerebral haemorrhage and blood–brain barrier (BBB) damage, a direct physical injury to the brain tissues that is difficult to prevent and usually irreversible, whereas secondary brain injuries, including calcium overload, oxidative stress, neuroinflammation and apoptosis, can be reversed^10^^,^ 11. A previous study^12^ reported that oxidative stress inhibition decreases mitochondrial apoptosis, improves neurological function and decreases cerebral oedema after TBI in mice; however, its neuroprotective effects remain unclear.

TBI occurs due to reactive oxygen species (ROS)-induced oxidative stress and glutamate-induced excitotoxicity, which can quickly result in neuron death in the brain^13^^,^ 14. ROS plays a fundamental role in neuron and tissue injuries and is generated by extra- or intracellular stimuli^15^. The molecular signalling pathway of oxidative stress is complex. Toll-like receptor 4 (TLR4), an innate immune receptor of bacterial endotoxins, is then accumulated to activate the nuclear factor kappa B (NF-κB) signalling transduction pathway, which was reported to be directly involved in inflammation and apoptosis in the epilepsy model^16^, myocardial infarction model^17^ and TBI^18^. As the brain tissue consumes more oxygen than most organs, ROS generation increases under oxidative stress after TBI^18^, subarachnoid haemorrhage^19^ or ischaemia-reperfusion injury^20^. Therefore, further studies of new potential drug targets in oxidative stress and apoptosis by targeting the TLR4/NF-κB/p65 signalling pathway are warranted.

Ulinastatin (UTI) is a serine protease inhibitor with a molecular weight of 67,000 purified from human urine. Its primary pharmacological activities were anti-inflammatory, immunoregulation and organ protection^21^^,^ 22. It is a drug widely used to treat acute inflammatory disorders, such as sepsis, ischaemia-reperfusion injury and antiapoptotic actions^23^. He *et al.* 24 reported that UTI may effectively inhibit postoperative increase in inflammatory agents and most likely provide pulmonary protective effects in cardiac surgery by a meta-analysis of 15 randomised controlled trials. Moreover, in recent animal studies, UTI had been reported to alleviate early brain injury (EBI), cerebral ischaemia-reperfusion injury and BBB permeability in the animal transient middle cerebral artery occlusion (tMCAO) model^25^^-^ 27. However, the effects of UTI on EBI in the acute phase of TBI are unclear, and their association with apoptotic molecular and oxidative stress levels remains to be elucidated.

In this study, a mouse TBI model was constructed to investigate the effects of UTI on EBI and explored the crosstalk between oxidative stress and apoptosis. Furthermore, the mechanism by which the TLR4/NF-κB/p65 signalling pathway may regulate this process was also explored.

## Methods

The study protocol was approved by the Anhui Medical University-Affiliated Wuxi Clinical College Clinical Research Ethics Committee (YXLL-2020-039).

All animal experiments performed in this study complied with the National Institutes of Health guidelines for the handling of laboratory animals and were approved by the Ethics Committee of the Wuxi Medical College of Anhui Medical University (YXLL-2020-039). Healthy adult male C57BL/6J mice (weighing 22–25g) (Anhui Medical University, Hefei, China) were used for all experiments. The mice were housed in animal care facilities on a 12-h light/dark cycle and had free access to food and water. A total of 45 mice were used and randomly assigned to the sham, TBI and TBI+UTI groups.

### Animal TBI model

The TBI model was established in strict accordance with the Feeney weight-drop model of focal injury^28^^,^ 29. Briefly, mice were anaesthetised by injecting 1% sodium pentobarbital (40 mg/kg) intraperitoneally and then placed in a brain stereotaxic apparatus. The rectal temperature was maintained at 37 ± 0.5 °C intraoperatively using a heating pad. Then, a burr hole was generated in the left hemisphere at the following coordinates: 0.2 mm posterior, 1 mm lateral and 2.2 mm below the horizontal plane of the bregma. The bone flap was removed to expose the dura mater, which was placed in a weight-drop device with an impact sensor. A metal (weight, 240 g; tip diameter, 3 mm) was inserted 1 cm above the dura mater through a catheter. Then, the scalp was closed, and the mice were removed from the apparatus. Finally, the hole was covered with medical bone wax. Animals in the sham group received similar surgical procedures but without the weight-drop impact.

### Drug administration

Ulinastatin (Techpool Biochem, Guangdong, China) was stored at 4 °C and dissolved in 0.9% normal saline when used. A 10^4^ U/kg of UTI was administered intraperitoneally before the onset of TBI^23^.

### Neurobehavioural assessment

The severity of brain injury was evaluated by determining neurological function 72 h after TBI using a previously described neurological grading system^30^. The scoring system consisted of motor, sensory, reflex and balance tests. The neurological scores ranged from 0 to 18 points and were calculated by adding the scores; all mice in each group underwent a behavioural assessment, and a higher score represented poor neurological functions. All behaviour scores of mice were recorded by the same independent observer who was blinded to the study groups.

### Brain water content measurement

The severity of brain oedema was evaluated by measuring the brain water content using the standard wet–dry method, as previously reported^31^^-^ 33. The mice were sacrificed 72 h after TBI, and the entire brain was harvested and separated into the ipsilateral and contralateral cortices, ipsilateral and contralateral basal ganglia and cerebellum (wet weight). Then, brain specimens from each group were dehydrated at 105 °C for 24 h to measure the dry weight. The percentage of brain water content was calculated by the formula in Eq. 1.


$$\frac{wet\;weight\;–\;dry\;weight\;}{wet\;weight}\;\times \;100$$ (1)

### Reactive oxygen species analysis

The nonfluorescent diacetylated 2´,7´-dichlorofluorescein probe (Sigma-Aldrich), which emits high fluorescence upon oxidation, was used to evaluate intracellular ROS production following the manufacturer’s instructions.

### Lipid peroxidation analysis

Malondialdehyde (MDA) levels were detected using a lipid peroxidation^34^ assay kit (Ex/Em 532/553 nm, Ab118970, Abcam, Cambridge, UK) according to the manufacturer’s instructions.

### Cytokine measurements of ipsilateral cortex tissues

The levels of interleukin (IL)-1β (cat. no. ab197742; Abcam), IL-6 (cat. no. ab222503; Abcam), tumour necrosis factor (TNF)-α (cat. no. ab208348; Abcam) and NF-κB (cat. no. ab176663; Abcam) were measured using enzyme-linked immunosorbent assay (ELISA) kits according to the manufacturer’s instructions.

### TUNEL staining

The TUNEL assay was performed to assess neuronal death in the hippocampus. The TUNEL reaction mixture (50 μL) was added to each sample, and the slides were incubated in a humidified chamber for 60 min at 37 °C in the dark. The slides were then incubated with 4´,6-diamidino-2-phenylindole for 5 min at room temperature in the dark to stain the nuclei, followed by imaging using a fluorescence microscope. The procedure was performed using a TUNEL staining kit following the manufacturer’s instructions. A negative control (without the TUNEL reaction mixture) was used.

### Western blot analysis

Western blot was performed as previously described^31^. Briefly, cerebral cortex samples were collected, homogenised and separated by sodium dodecyl sulphate-polyacrylamide gel electrophoresis on 10% polyacrylamide gels. A BCA Protein Assay Kit (Beyotime) was used to measure protein concentrations using the bicinchoninic acid method. After separation, protein samples were transferred onto immobilon nitrocellulose membranes. The membranes were blocked with 5% nonfat milk at room temperature for 1 h and then incubated with the following primary antibodies overnight at 4 °C: rabbit anti-β-actin (1:1000, rabbit polyclonal, Abcam, ab8227), rabbit anti-caspase-3 (1:2000, rabbit polyclonal, Abcam, ab184787), rabbit anti-Bax (1:2.000, ab182733), rabbit anti-Bcl2 (1:2.000, ab182858), rabbit anti-TLR4 (1:1000, rabbit polyclonal, Abcam, ab13556) and rabbit anti-NF-κB p65 (1:1000, rabbit monoclonal, Abcam, ab32536). After washing the membranes with TBST three times, horseradish peroxidase-conjugated goat antirabbit immunoglobulin G (IgG) or goat antimouse IgG secondary antibodies (1:5000) were used, and the membranes were incubated using secondary antibodies at room temperature for 1.5 h. The protein bands were detected using a Bio-Rad imaging system (Bio-Rad, Hercules, CA, USA) and quantified with ImageJ software.

### Quantitative reverse transcription polymerase chain reaction

Quantitative reverse transcription polymerase chain reaction (qRT-PCR) was performed as indicated previously^35^. Total RNA was extracted from hippocampal brain samples using TRIzol Reagent (Gibco; Thermo Fisher Scientific, Inc., Waltham, MA, USA) following the manufacturer’s instructions. Thereafter, the RNA was reverse transcribed to complementary DNA (cDNA) using the RevertAid First Strand cDNA Synthesis Kit (K1622; Thermo Fisher Scientific Inc., Rockford, IL). TLR4 and NF-κB mRNA levels in each sample were measured with qPCR using SYBR Green Master Mix (Toyobo Co., Ltd., Osaka, Japan). Glyceraldehyde 3-phosphate dehydrogenase (GAPDH) was used as an internal control. The qPCR thermocycling conditions were as follows: 45 °C (2 min) and 95 °C (10 min), followed by 40 denaturation cycles at 95 °C (15 s), annealing at 60 °C (1 min) and extension at 72 °C (1 min). All samples were analysed in triplicate. The target genes and specific primers are as follows:

- TLR4 (forward, 5´-GACGTGGAACTGGCAGAAGA-3´; reverse, 5´-ACTGATGAGAGGGAGGCCAT-3´),
- NF-κB (forward, 5´-GCGAGAGAAGCACAGATACCA-3´; reverse, 5´-GGTCAGCCTCATAGTAGCCA-3´)
- GAPDH (forward, 5´-ATGGGTGTGAACCACGAGA-3´ and reverse, 5´-CAGGGATGATGTTCTGGGCA-3´)

### Statistical analysis

Data were expressed as means and standard error of the mean (SEM). The Statistical Package for the Social Sciences 14.0 (SPSS, Chicago, IL, USA) and GraphPad Prism 6 (GraphPad Software, San Diego, CA, USA) software were used for statistical analyses. Student’s t-test was used to compare two groups, and one-way analysis of variance (ANOVA), followed by Bonferroni’s post-hoc test, was used to compare two independent variables. For no normally distributed data and/or data with a nonhomogeneous variance, the Kruskal–Wallis test, followed by Dunn’s post-hoc test, was used. For all statistical analyses, differences were considered significant at p < 0.05.

## Results

### Ulinastatin alleviates neurological deficits and brain oedema after TBI

**Figure 1:**
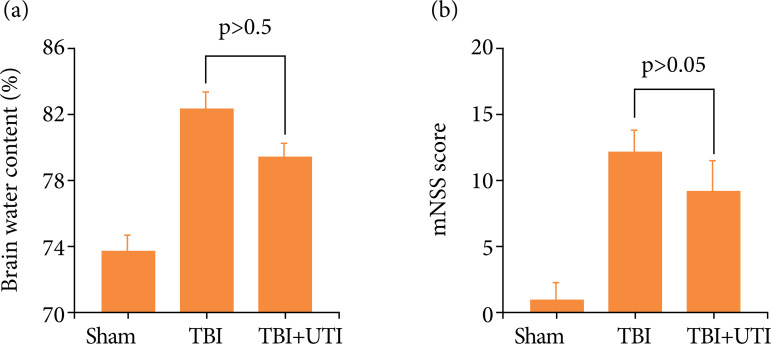
Ulinastatin alleviates neurological deficits and brain oedema after TBI. **(a)** Comparison of the brain water content among the three groups (n = 5, p < 0.05). **(b)** Neurological scores of mice in the sham, TBI and TBI groups treated with UTI at 72 h after TBI (n = 10, p < 0.05). ANOVA; means ± SEM.

The modified neurological severity score was calculated to evaluate neurological deficits and determine the brain water content using the wet–dry method at 72 h after TBI to evaluate brain damage and clarify the neuroprotective effects of UTI on TBI. TBI significantly increased the brain water content, which was alleviated by the UTI treatment (Fig. 1a). Similar results were obtained for neurological scores, which were significantly decreased after TBI, and UTI administration significantly improved the neurological function (Fig. 1b).

### Ulinastatin alleviates hippocampal cell apoptosis after TBI

**Figure 2:**
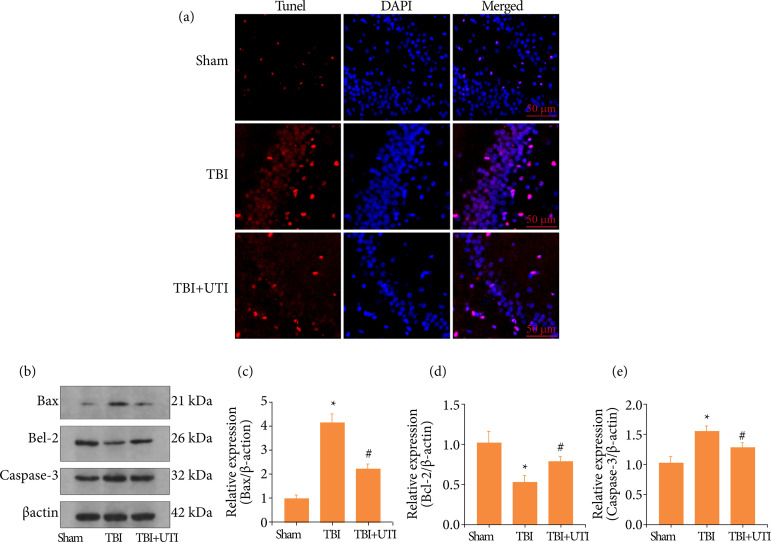
Ulinastatin alleviates neuronal apoptosis after TBI. **(a)** TUNEL staining showed that UTI alleviated neuronal apoptosis in the hippocampus at 72 h after TBI, and representative images of apoptotic neurons are shown. Scale bar = 50 μm. DAPI, 4´,6-diamidino-2-phenylindole; SAH, subarachnoid haemorrhage; TUNEL, terminal deoxynucleotidyl transferase dUTP nick end labelling. **(b)** Levels of caspase-3, Bcl-2 and Bax in the brain cortex of mice after TBI were determined using Western blotting. (c-e): Quantification of caspase-3, Bcl-2 and Bax in the brain cortex relative to b-actin, the loading control. UTI reduced Bax **(c)** and caspase-3 **(e)** levels in mice with TBI and increased the Bcl-2 level **(d)**. (n = 5, *p < 0.05 vs. the sham group; #p < 0.05 vs. the TBI group; ANOVA; means ± SEM).

Hippocampal cell apoptosis is the primary factor that resulted in EBI after TBI. Therefore, a TUNEL assay was used to evaluate cell death in TBI mice treated with and without UTI at 72 h after model construction. Expression levels of apoptosis-related proteins were also detected using western blotting. The results revealed more hippocampal cell death after TBI, and UTI decreased cell apoptosis (Fig. 2a). Apoptosis-related protein caspase-3, Bcl-2 and Bax were detected via western blotting (Fig. 2b). Apoptosis-induced proteins, Bax (Fig. 2c) and caspase-3 (Fig. 2e) were significantly decreased after UTI treatment, whereas apoptosis inhibited protein and Bcl-2 (Fig. 2d) significantly increased after the UTI administration. Based on these results, UTI exerts the neuroprotective effects after TBI.

### Ulinastatin alleviates neuroinflammation after TBI

**Figure 3:**
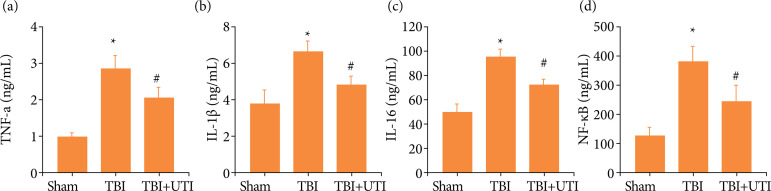
Ulinastatin alleviates neuroinflammation post TBI. UTI significantly reduced hippocampal **(a)** TNF-a, **(b)** interleukin-1β (IL-1β), **(c)** IL-6 and **(d)** NF-kB levels 72 h after TBI (n = 5, ^*^p < 0.05 vs. the sham group; ^#^p < 0.05 vs. the TBI group, ANOVA; means ± SEM).

As previous studies identified a vital role for neuroinflammation in EBI after TBI, increased neuroinflammation aggravates EBI^1^^-^ 4. The inflammatory complex induces secretion of proinflammatory cytokines, including IL-1β, IL-6 and TNF-a, and the subsequent activation of proinflammatory signalling through the NF-kB to initiate apoptosis. Therefore, the hippocampal levels of IL-1β, IL-6, TNF-a and NF-kB were measured using ELISAs. The proinflammatory cytokine levels were significantly increased after TBI but significantly decreased after UTI treatment (Fig. 3a-d). Hence, these results suggested that UTI exhibited potent anti-inflammatory activities against TBI-induced neuroinflammation.

### Ulinastatin inhibits TBI-induced oxidative stress in the hippocampus

**Figure 4:**
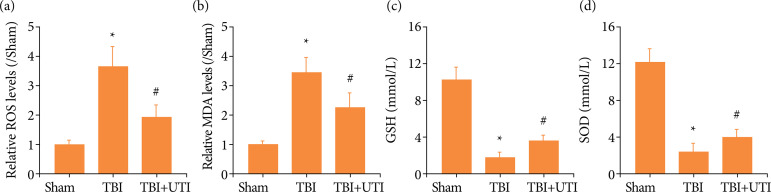
Ulinastatin inhibits TBI-induced oxidative stress in the hippocampus. **(a)** The ROS level by the DCF-DA method. (b-d) The MDA, GSH and SOD levels were quantified using commercial kits (n = 5, data are presented as the mean ± SEM, ^*^p <0.05 vs. the sham group; ^#^p < 0.05 vs. the TBI group).

To understand the neuroprotective mechanisms of UTI, to clarify whether oxidative stress plays an important role in TBI and the regulatory effects of UTI. Oxidative stress was activated and a large amount of ROS was produced after TBI. ROS and MDA were vital oxidative stress biomarkers. ROS, glutathione, superoxide dismutase (SOD) and MDA levels were detected (Fig. 4a-d). Both ROS and MDA levels were increased after TBI but significantly decreased after UTI treatment, as GSH and SOD levels were decreased after TBI but significantly increased after UTI treatment. Therefore, these results showed that UTI inhibited oxidative stress activation after TBI.

### Ulinastatin regulates oxidative stress and apoptosis by modulating the TLR4/NF-kB/p65 signalling pathway after TBI

**Figure 5:**
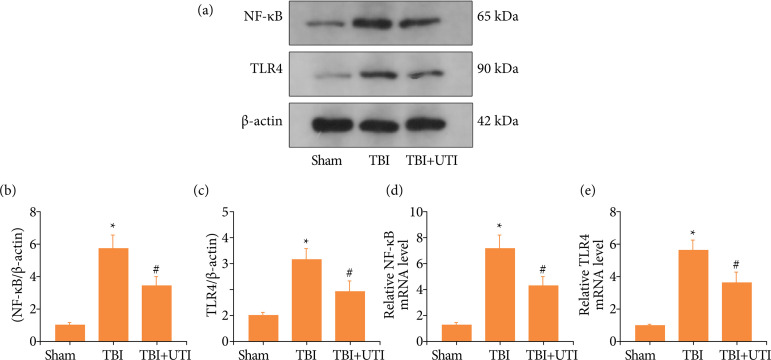
Ulinastatin regulates oxidative stress and apoptosis by modulating the TLR4/NF-kB/p65 signalling pathway after TBI. **(a)** TLR4 and NF-kB levels in the brain cortex of mice after TBI were determined using western blotting; **(b)** Quantification of NF-kB levels in the brain cortex relative to β-actin, the loading control; **(c)** Quantification of TLR4 levels in the brain cortex relative to β-actin; **(d)** NF-kB mRNA levels in the brain of TBI mice were measured by real-time PCR; **(e)** TLR4 mRNA levels in the brain of TBI mice were measured by real-time PCR (n = 5, data are presented as the mean ± SEM, ^*^p < 0.05 vs. the sham group; ^#^p < 0.05 vs. the TBI group).

TLR4/NF-kB/p65 is a core signalling pathway of oxidative stress and apoptosis. Previous studies have demonstrated that activation of the TLR4/NF-kB/p65 signalling pathway is partially dependent on ROS production^5^^,^ 6. Therefore, it was important to explored whether the neuroprotection of UTI regulates oxidative stress and apoptosis by modulating the TLR4/NF-kB/p65 signalling pathway after TBI in this study. TLR4 and NF-kB protein levels was detected by performing western blotting (Fig. 5a). The TLR4 and NF-kB/p65 levels were significantly increased in the TBI group and decreased after UTI administration (Fig. 5b and c). Additionally, qRT-PCR demonstrated similar results (Fig. 5d and e). Thus, these results showed that neuroprotection of UTI may regulate the TLR4/NF-kB/p65 signalling pathway.

## Discussion

**Figure 6:**
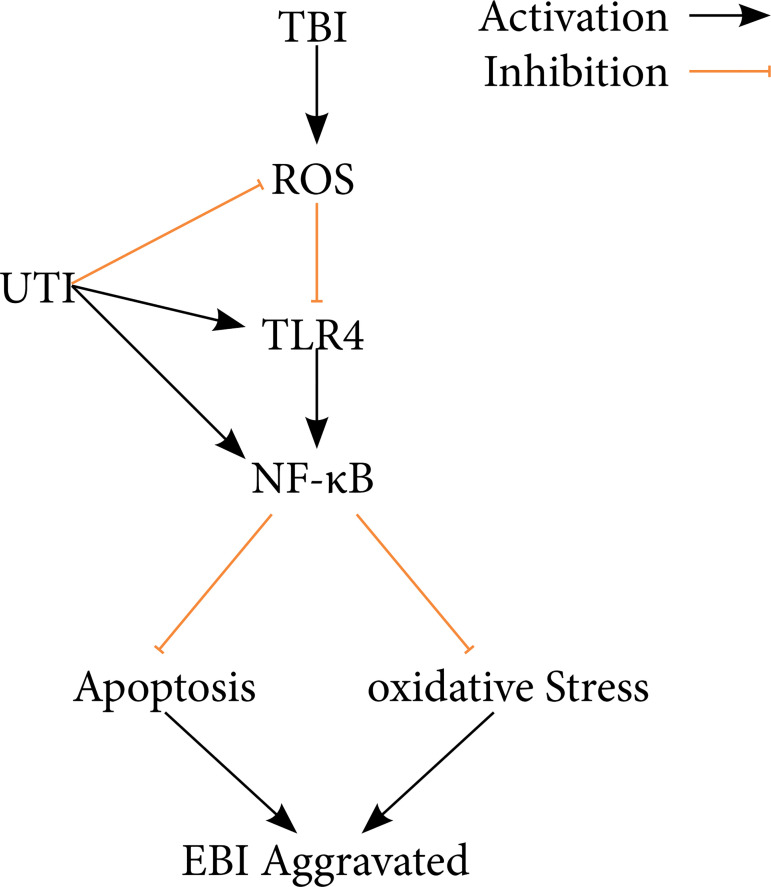
A diagram of the proposed model explaining the observations of TLR4/NF-kB/p65-mediated regulation of oxidative stress, neuroinflammation and apoptosis after TBI and potential mechanisms underlying the effects of the UTI intervention.

In this study, the therapeutic potential of UTI to alleviate EBI in a TBI mouse model was evaluated. UTI is a neuroprotective agent that attenuates EBI after TBI. The results showed that UTI (1) improves neurological dysfunction after TBI, (2) alleviates brain damage in a mouse TBI model, (3) relieves neuroinflammation after TBI, decreases inflammatory brain damage, (4) prevents oxidative stress and apoptosis after TBI and alleviates neuronal death and (5) prevents the antiapoptosis and antioxidative stress effects of UTI that may be related to the TLR4/NF- kB/p65 signalling pathway (Fig. 6).

Ulinastatin is a 67 kDa glycoprotein purified from the urine of healthy humans, nonspecific protease inhibitor and a urinary trypsin inhibitor, which is used to treat acute inflammatory disorders, sepsis, toxic shock and haemorrhagic shock^7^^,^ 8.Recent studies demonstrated that UTI can alleviate cerebral ischaemia-reperfusion injury by regulating inflammation and oxidative stress^9^^,^ 10. However, the neuroprotection of UTI in TBI was unclear and lacked related clinical studies. Liu *et al.* 11 reported that UTI can significantly decrease the brain water content and BBB permeability after TBI, which may be caused by decreased activation of astrocytes and ET-1 and inhibited expression of proinflammatory VEGF and MMP-9. Another study also reported that UTI can attenuate brain oedema in male Sprague-Dawley rats with TBI, with the preliminary molecular mechanism may be through decreased expression level of aquaporin-4 (AQP4) and proinflammatory cytokines, such as IL-1β and TNF-α and NF-κB activity^12^. In the present study, UTI was found to alleviate brain oedema, improve neurological function, relieve neuroinflammation response and decrease hippocampal neuronal damage and apoptosis.

The pharmacological action of UTI was more complex, including anti-inflammatory, immunoregulation and organ protection^11^^,^ 13. The neuroprotection mechanism of UTI after TBI also varied and complicated. Cui *et al.* 9 reported that UTI can ameliorate cerebral ischaemia-reperfusion (I/R) injury by regulating inflammation and oxidative stress, the pharmacological mechanism of UTI that may mediate the Nrf-2/HO-1 signalling pathway. Li *et al.* 14 also demonstrated that UTI can protect the brain against ischaemic injury, with the potential molecular mechanism through the restoration of the BBB permeability by decreasing the MMP-9 expression and increasing ZO-1 and occludin protein expressions. Based on the present data, preliminary results showed that UTI can inhibit oxidative stress, neuroinflammation and apoptosis. Koga *et al.* 15 also confirmed that UTI can suppress ROS generation, oxidative stress and early inflammation in the forebrain I/R of mice. Furthermore, UTI also can inhibit hippocampus endoplasmic reticulum stress and apoptosis after acute paraquat poisoning^16^. Anti-apoptosis, anti-neuroinflammation and antioxidative stress of UTI in central nervous system diseases also were confirmed^10^^,^ 17 ^,^ 18.

The mechanisms and molecules regulating oxidative stress and apoptosis were complex and involved NF-kB pathways. The results of this study revealed that UTI decreases the ROS production, subsequently inhibiting the activation of oxidative stress, and UTI also can increase the TLR4 and NF-kB/p65 expression levels and then alleviate the activation of oxidative stress and apoptosis. UTI can activate the TLR4/JNK pathway; inhibit the expression of downstream targets in the TLR4/JNK pathway, such as NF-kB (p-p65), and apoptosis-related proteins; and then alleviate the oxidative stress, neuroinflammation and apoptosis^19^. Li *et al.* 20 also reported that UTI can improve neurological function and alleviate brain oedema and infarct volume by decreasing the TLR4 and NF-κB expressions in the tMCAO model. A recent study also indicated that MCAO mice treated with a nanoparticle formulation of TLR2shRNA- and TLR4shRNA (T2sh+T4sh)-expressing plasmids can increase the expression of both TLRs 2 and 4 and their downstream signalling molecules including the proinflammatory cytokines, alleviate acute inflammation and improve neurological recovery^21^. Cui *et al.* 12 confirmed that IL-1β, TNF-α and NF-κB activities were inhibited by UTI treatment in TBI, which resulted in intracellular ROS accumulation and decrease TLR4 expression levels, and the TLR4/NF-kB/p65 signalling pathway also directly regulates oxidative stress and apoptosis. The specific mechanism remains unclear, and other potential molecular mechanisms may play important roles. Therefore, further studies are needed to explore these mechanisms.

Furthermore, this experiment was performed in mice, and whether the treatment is effective in humans remains controversial. Oxidative stress and neuroinflammation were the main reasons that lead to brain injury in patients with TBI and immediately after the occurrence of TBI. It supposes that early UTI intervention can prevent brain injury and improve neurological deficits and outcomes in patients with TBI. In the future, it will further explore the clinical effects of UTI in patients with TBI.

## Conclusions

This study provided evidence that oxidative stress and apoptosis are mediated by ROS, which emerged as an important cellular regulatory mechanism and contributed to EBI after TBI. Moreover, UTI-mediated regulation of oxidative stress and apoptosis through the TLR4/NF-kB/p65 pathway and provided a new idea to explore the biological effects and mechanisms underlying the anti-oxidative stress, anti-inflammatory, anti-apoptosis and neuroprotective properties of UTI.
